# Plant Salinity Stress Response and Nano-Enabled Plant Salt Tolerance

**DOI:** 10.3389/fpls.2022.843994

**Published:** 2022-03-22

**Authors:** Zengqiang Li, Lan Zhu, Fameng Zhao, Jiaqi Li, Xin Zhang, Xiangjun Kong, Honghong Wu, Zhiyong Zhang

**Affiliations:** ^1^MOA Key Laboratory of Crop Ecophysiology and Farming System in the Middle Reaches of the Yangtze River, College of Plant Science and Technology, Huazhong Agricultural University, Wuhan, China; ^2^Henan Collaborative Innovation Centre of Modern Biological Breeding, Henan Institute of Science and Technology, Xinxiang, China; ^3^Shenzhen Institute of Nutrition and Health, Huazhong Agricultural University, Shenzhen, China; ^4^Shenzhen Branch, Guangdong Laboratory for Lingnan Modern Agriculture, Genome Analysis Laboratory of the Ministry of Agriculture, Agricultural Genomics Institute at Shenzhen, Chinese Academy of Agricultural Sciences, Shenzhen, China

**Keywords:** mechanisms, nanomaterials, phytohormones, reactive oxygen species, salt tolerance, Na^+^/K^+^ homeostasis

## Abstract

The area of salinized land is gradually expanding cross the globe. Salt stress seriously reduces the yield and quality of crops and endangers food supply to meet the demand of the increased population. The mechanisms underlying nano-enabled plant tolerance were discussed, including (1) maintaining ROS homeostasis, (2) improving plant’s ability to exclude Na^+^ and to retain K^+^, (3) improving the production of nitric oxide, (4) increasing α-amylase activities to increase soluble sugar content, and (5) decreasing lipoxygenase activities to reduce membrane oxidative damage. The possible commonly employed mechanisms such as alleviating oxidative stress damage and maintaining ion homeostasis were highlighted. Further, the possible role of phytohormones and the molecular mechanisms in nano-enabled plant salt tolerance were discussed. Overall, this review paper aims to help the researchers from different field such as plant science and nanoscience to better understand possible new approaches to address salinity issues in agriculture.

## Introduction

Salinity is a main stress-limiting agricultural production. Feeding over 9.3 billion populations in 2050 is a big challenge. It is estimated that in 2050, agricultural production needs to be increased over 60% at the 2005–2007 level (Fita et al., 2015). However, efficient agricultural production is always threatened by stress conditions such as salinity. In recent years, climate change, seawater backflow, groundwater infiltration, and human-being activities such as irrigation and fertilizer application increased salt concentration in soil, resulting in soil salinization (Zhang et al., 2016; Qian et al., 2021). Soil salinization inhibits plant growth, yield, and product quality (Yang and Guo, 2018a). While more than 950 million hectares of land are affected by salinity stress, the trend of soil salinization is increasing (Yang and Guo, 2018b).

The main components of salt stress in plants are osmotic stress, ionic stress, and secondary stress, i.e., ROS over-accumulation (Parihar et al., 2015; Morton et al., 2018). Firstly, upon the onset of salt stress, high salinity reduces the water potential around the plant roots, limiting root absorption of water (Negrão et al., 2016). Secondly, over-accumulation of sodium and chloride in plants causes ion toxicity. It not only disrupts ion homeostasis such as Na^+^ and K^+^ homeostasis (Zhu, 2002), but also hinders the efficient uptake of nutrient elements such as Ca^2+^, resulting in the lack of essential nutrients in plants (Zhang et al., 2017; Wu, 2018a; Li et al., 2021). Osmotic and ionic stresses lead to over-accumulation of reactive oxygen species (ROS) in plants, resulting in oxidative stress (Zhu, 2016; Wu et al., 2018b). For example, excessive superoxide anion (O_2_^−^) and hydrogen peroxide (H_2_O_2_) are accumulated in chloroplasts and mitochondria, affecting photosynthesis and respiration of plants under salt stress (Balal et al., 2011). Moreover, the structure of macromolecules such as DNA and protein can be damaged by excessive ROS (Hu et al., 2021; Liu et al., 2021a). Nowadays, besides genetic engineering and exogenous application of antioxidants, nanomaterials showed good potential in improving plant salt tolerance although the underlying mechanisms are less addressed. Nano-enabled plant salt tolerance could be an alternative approach to help to enable efficient agricultural production.

In this review, we summarized the molecular mechanisms underlying plant salt tolerance and emphasized the importance of nanotechnology in improving plant salt tolerance. We hope this review will set up an idea to help the researchers in plant science and nanoscience to better understand possible new approaches to address issues such as salinity in agriculture.

## Use of Nanomaterials: an Emerging Approach to Improve Plant Salt Tolerance

**Figure 1:**
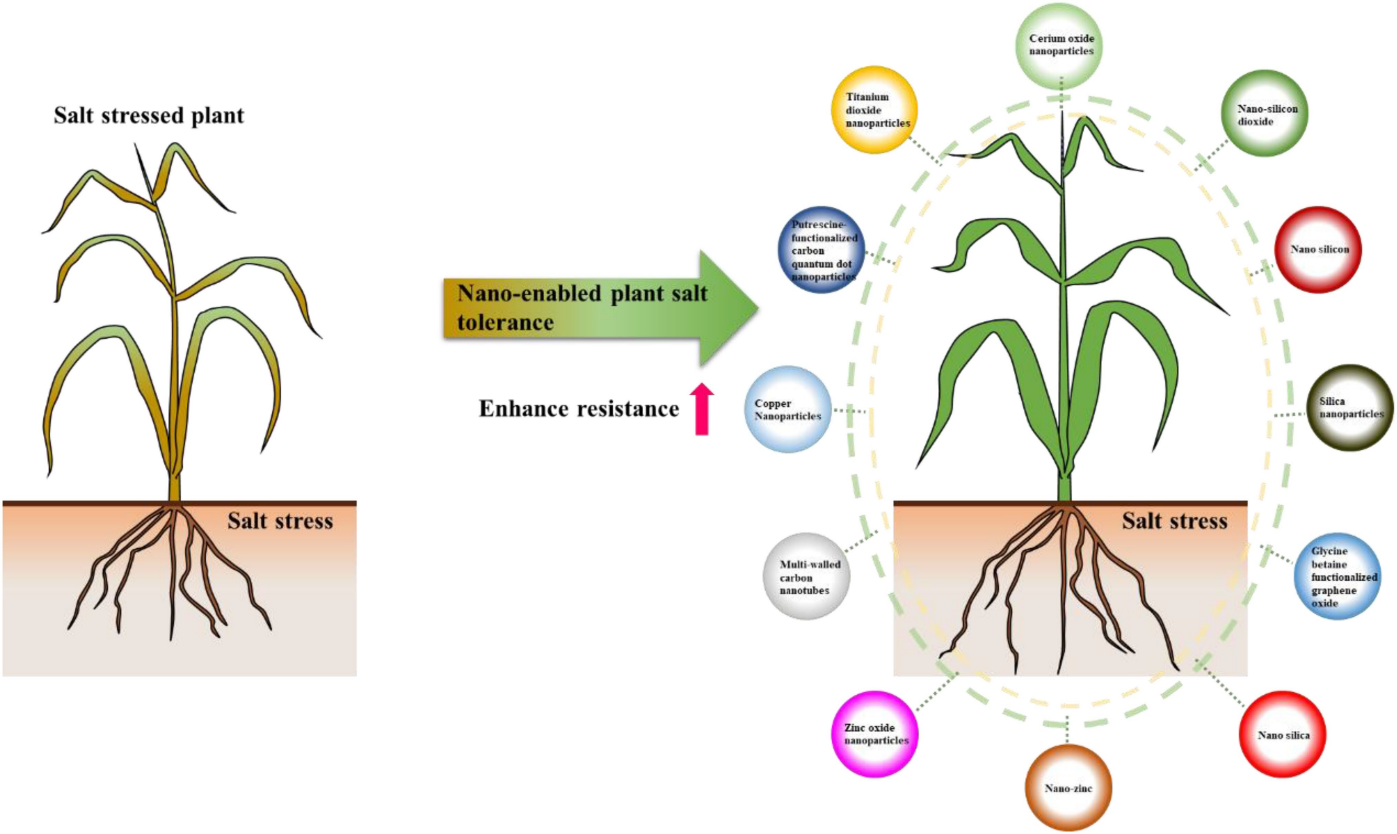
The used nanomaterials for improving plant salt tolerance.

In recent years, plant nano-biotechnology approach showed good potential to improve plant stress tolerance. Nano-enabled agriculture is a hot research topic. Nanotechnology refers to the technology of manipulating materials with a basic structure of 1–100 nm in at least one dimension (Farokhzad and Langer, 2009). The history of adoption of nanotechnology in agriculture is relatively short, but it showed great potential in agriculture, such as the development of nano-fertilizer, nano-pesticides, and smart plant construction (Giraldo et al., 2019; Kah et al., 2019). As an emerging strategy to promote agricultural production, plant nanotechnology shows good potential in agriculture, such as seed treatment and germination, plant growth and development, pathogen diagnosis, genetic engineering, plant stress tolerance, crop nutrition, and detection of toxic agrochemicals (Farooq et al., 2012; Bui, 2013; Kah et al., 2019). In recent years, use of nanomaterials (NMs) to enhance plant stress tolerance showed the potential to become an economical, effective, and sustainable strategy for efficient agricultural production. NMs enhance plant tolerance to salt by protecting plant photosynthesis, enabling ROS detoxification, and alleviating osmotic and ionic stress (Gao et al., 2007; Rico et al., 2015; Khan et al., 2016a). Nano-enabled plant salt tolerance has been reported in many species, including *Arabidopsis*, wheat, cotton, and so on (Mushtaq et al., 2017; Michael et al., 2018; Liu et al., 2021b). To date, the used nanomaterials which improved plant salt tolerance include silica nanoparticles, cerium oxide nanoparticles, put-carbon quantum dots (put-CQD) nanoparticles, titanium dioxide nanoparticles, carbon nanotubes, and nano-zinc (Figure 1). For example, multi-walled carbon nanotubes (MWCNTs), cerium oxide nanoparticles, and zinc oxide nanoparticles (SeNPs and ZnONPs) can significantly alleviate the inhibition of salt stress on the growth of rapeseed seedlings. Seed priming using cerium oxide nanoparticles, SeNPs and ZnONPs, also significantly improves the germination rate of rapeseed seeds under salt stress (Rossi et al., 2017; Zhao et al., 2019; El-Badri et al., 2021; Khan et al., 2021; Li et al., 2022). For more details, please refer to Table 1.

**Table 1:** The known nanomaterials used for improving plant salt tolerance.

Crop species	Nanomaterials	Dosage	Size and zeta potential	Reference
*Arabidopsis*	Cerium oxide nanoparticles	50 mg/L	10 nm, −17 mV	Michael et al., 2018; Wu et al., 2018c
Bean	Titanium dioxide nanoparticles	0.01%	20–30 nm	Abdel-Latef et al., 2017
*Brassica napus*	Cerium oxide nanoparticles	0.5 mg/kg	52.6 nm, −51.8 mV	Rossi et al., 2017
*Brassica napus*	zinc oxide nanoparticles	100 mg/L	10–55 nm, −32.4 mV	El-Badri et al., 2021
Cotton	Nano-zinc	100 and 200 ppm	Not reported	Hussein and Abou-Baker, 2018
Cotton	Cerium oxide nanoparticles	0.9 mM	6.05 nm, −15.30 mV	Liu et al., 2021b
Cucumber	Silica nanoparticles	200 ppm	10 nm	Alsaeedi et al., 2018
*Cucurbita pepo*	Nano-silicon dioxide	6.0 mM	10 nm	Siddiqui et al., 2015
Grapevine	Putrescine-functionalized carbon quantum dot (put-CQD) nanoparticles	10 mg/L	Not reported	Gohari et al., 2021
Rapeseed	Cerium oxide nanoparticles	0.1 mM	8.6 nm, −25.3 mV	Khan et al., 2021; Li et al., 2022
Rapeseed	Multi-walled carbon nanotubes (MWCNTs)	0.1 mg/ml	Not reported	Zhao et al., 2019
Rice	Nano silica	150 g/L	20–30 nm	Abdel-Haliem et al., 2017
Soybean	Nano-sillicon	1 mM	20–30 nm	Farhangi-Abriz and Torabian, 2018
Strawberry	Nano-Silicon Dioxide	50 mg/L	10–20 nm	Avestan et al., 2019
Sweet basil	Glycine betaine functionalized graphene oxide	50 mg/L	Not reported	Ganjavi et al., 2021
Sweet pepper	Nano silicon	1.0 cm^3^/L	Not reported	Tantawy et al., 2015
Tomato	ZnO nanoparticles	50 mg/L	Not reported	Faizan et al., 2021
Tomato	Copper nanoparticles	250 mg/L	20–50 nm	Pérez-Labrada et al., 2019
Wheat	Silica nanoparticles	50 nM	50 nm, 100 nm	Mushtaq et al., 2017

**Figure 2:**
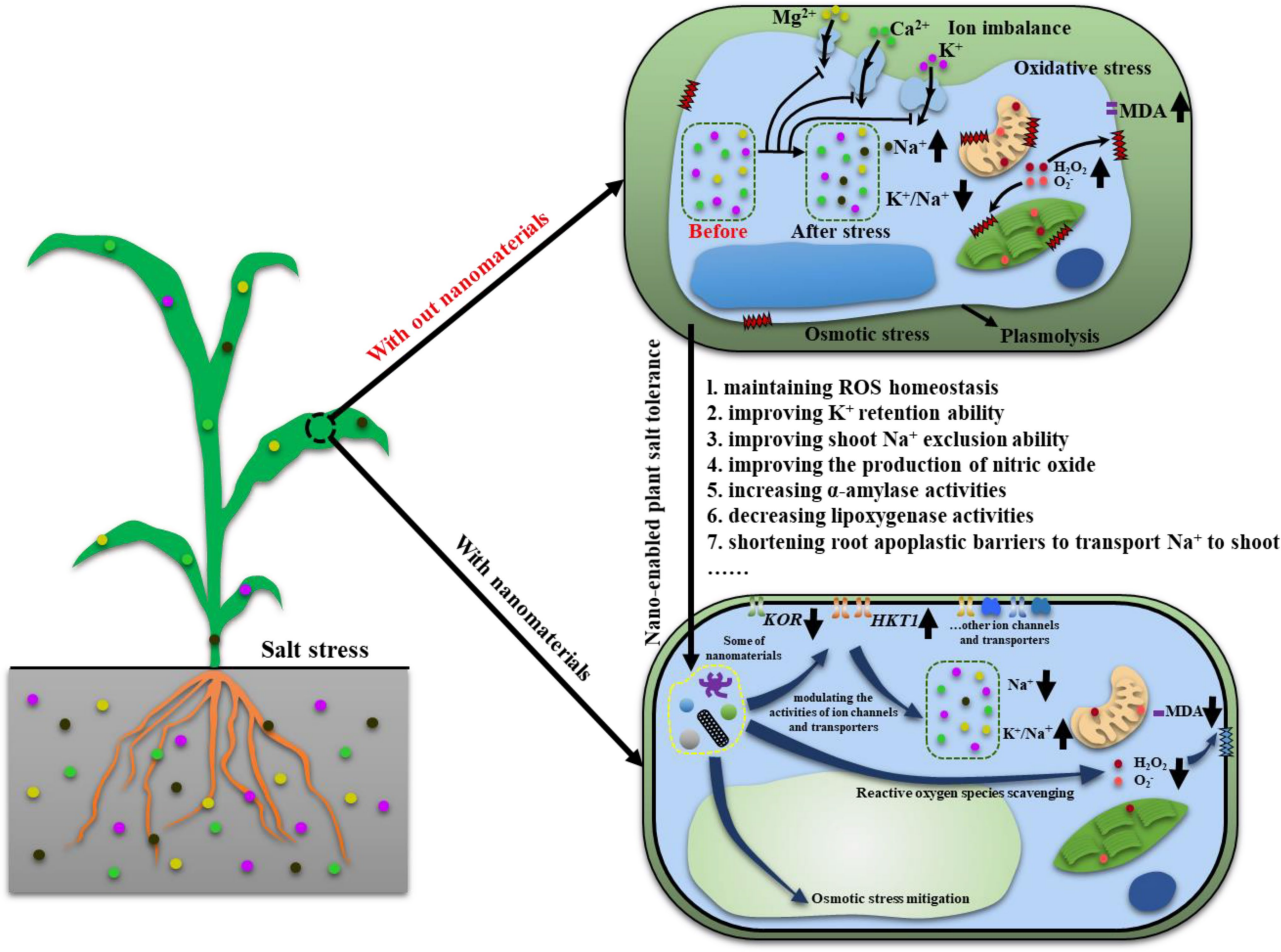
A model showing how nanomaterials can help with maintaining osmotic balance, ion homeostasis and ROS homeostasis.↑: increase, ↓: decrease, ┤: inhibition.

As mentioned in Table 1, many nanomaterials are used to improve plant salt tolerance. Among them, cerium oxide nanoparticles (nanoceria) are one of the widely used nanomaterials. Thus, here, we used nanoceria as example to discuss how nanomaterials can help to improve plant salt tolerance. Nanoceria are known as nanozyme and potent catalytic ROS (reactive oxygen species) scavenger, having a large number of surface oxygen vacancies which can convert ROS, i.e., H_2_O_2_, O_2_^−^, and ^•^OH to its non-radical counterparts. To date, the mechanisms behind nanoceria improved plant salt tolerance (Figure 2) are: (1) maintaining ROS homeostasis *via* direct scavenging of ROS or modulating antioxidant system (Rossi et al., 2017; Wu et al., 2018c), (2) improving mesophyll cells’ ability to retain K^+^ (Wu et al., 2018d), (3) improving shoot Na^+^ exclusion ability to avoid over-accumulation of Na^+^ in leaf (Liu et al., 2021a), (4) improving the production of gas signaling molecules, i.e., NO (nitric oxide; Zhou et al., 2021), (5) increasing α-amylase activities to improving seed germination (Khan et al., 2021), (6) decreasing lipoxygenase activities to reduce membrane oxidative damage (Li et al., 2022 ES Nano), and (7) allowing Na^+^ being transported to shoot *via* shortening root apoplastic barriers (Rossi et al., 2017). Some of these mechanisms might be shared between different nanomaterials in terms of improving plant salt tolerance. For example, Mn_3_O_4_ nanoparticles scavenged over-accumulated ROS to improve salt tolerance in cucumber (Lu et al., 2020). Zinc oxide nanoparticles modulated the activities of antioxidant enzymes to help to maintain ROS homeostasis in tomato plants (Faizan et al., 2021). These results suggested that in different nanomaterials can execute similar role to improve salt tolerance in varied plant species. Further, MWCNTs increased rapeseed plants’ ability to tolerate salinity by maintaining Na^+^/K^+^ ratio (Zhao et al., 2019). This is similar to the findings that nanoceria can help to maintain Na^+^/K^+^ ratio to improve cotton salt tolerance (Liu et al., 2021a), further confirming that some common mechanisms might be employed in nano-enabled plant salt tolerance. More efforts are needed to investigate what are the commonly employed mechanisms in nano-enabled plant salt tolerance and what are the mechanisms specially associated with one or few types of nanomaterials.

## Plant’s Ability to Alleviate Oxidative Stress is Important for Salt Tolerance

Reactive oxygen species (ROS) mainly include superoxide anion (O_2_^−^), hydrogen peroxide (H_2_O_2_), hydroxyl radical (•OH), and singlet oxygen (^1^O_2_). Under normal conditions, there is a dynamic balance between the production and scavenging of ROS in plant cells. When plants are subjected to salt stress, apoplast and organelles such as chloroplasts and mitochondria accumulate excessive ROS, which in turn results in lipid peroxidation and damage of the structure of DNA and protein, causing oxidative stress (Mignolet-Spruyt et al., 2016; Wang et al., 2017; Jia et al., 2020). ROS scavenging in plants mainly involves the antioxidant enzyme system and the non-enzymatic system. The former one mainly includes superoxide dismutase (SOD), peroxidase (POD), catalase (CAT), ascorbate peroxidase (APX), and glutathione peroxidase (GPX), and the latter one mainly includes ascorbic acid, glutathione, vitamin E, carotenoid, and mannitol (Deinlein et al., 2014; Liang et al., 2017; Irshad et al., 2021; Li et al., 2021). SOD mainly catalyzes O_2_^−^ to produce H_2_O_2_ and O_2_. This process was often regarded as the first defense for plants to scavenge excessive ROS (Zhu, 2002, 2016). POD, CAT, and APX mainly scavenge H_2_O_2_ accumulated in plants (Petridis et al., 2012; Hou et al., 2016; Liu et al., 2020). After being subjected to salt stress, the activity of antioxidant enzymes in salt tolerant plants is generally higher than the sensitive ones (Zhu, 2002, 2016; Wu et al., 2020). Overexpression of *GhSOD1* and *GhCAT* significantly improves salt tolerance of cotton (Luo et al., 2000). Similarly, applying nanozymes with ROS scavenging ability in plants helped to improve plant salt tolerance *via* maintaining ROS homeostasis (Wu, 2018a; Wu et al., 2018c; Zhao et al., 2020). For example, nanozyme poly(acrylic) acid-coated cerium oxide nanoparticles (PNC) can improve salinity stress tolerance of cotton mainly by alleviating ROS accumulation in seedling roots (An et al., 2020). Similar results were also found in wheat (Mushtaq et al., 2017), tomato (Faizan et al., 2021), soybean (Farhangi-Abriz and Torabian, 2018), and rapeseed (Khan et al., 2021). Carbon quantum dots can significantly improve the activity of antioxidant enzyme system to reduce the content of ROS and to alleviate oxidative damage on cell membrane, thus enhancing grape salt tolerance (Gohari et al., 2021). Cucumber plants with foliar-delivered Mn_3_O_4_ nanoparticles showed less ROS accumulation and better salt tolerance than the control without nanoparticles (Lu et al., 2020). Spraying zinc oxide NMs on leaves can also significantly improve the protein content and antioxidant enzyme activity of POX, SOD, and CAT in tomato plants under salt stress (Faizan et al., 2021). It was found that compared with salt control, zinc oxide NMs upregulated the expression levels of SOD and GPX genes in tomato under salt stress (Alharby et al., 2016). Thus, improving the ability to maintain ROS homeostasis could be one of the important mechanisms under nano-enabled plant salt tolerance.

Nanomaterials might also improve plant salt tolerance *via* modulating the production of antioxidants. Glutathione, proline, and ascorbic acid are important soluble antioxidants in plant cells, playing an important role in maintaining ROS homeostasis in cells (Zhu, 2002). (1) Reduced glutathione (GSH) is a widely distributed antioxidant in cells. The dynamic balance between GSH and oxidized glutathione is also an important indicator of the antioxidant capacity of plant cells (Hameed et al., 2014). Indeed, exogenously applied GSH can improve salt tolerance in many plant species such as onion (Hamed and Al-Mutawa, 2009), tomato (Zhou et al., 2017), and mung bean (Nahar et al., 2015). To our surprise, to date, the role of GSH and how it works in nano-enabled plant salt tolerance is rarely investigated. CuNPs improved tomato salt tolerance *via* increasing the content of glutathione (Pérez-Labrada et al., 2019). Future studies are encouraged to investigate the role of GSH and its biosynthesis in nano-enabled plant salt tolerance. (2) Proline is known as one of the most important and effective organic osmotic regulatory substances, which plays an vital role in maintaining osmotic balance and cell membrane integrity (Siddiqui et al., 2015; Hu et al., 2021). Besides being effective osmotic regulatory substance, proline is also known as antioxidants to scavenge ROS (Siddiqui et al., 2015; Brahimova et al., 2021; Hu et al., 2021). Proline is often used as an important physiological indexes in plant salt tolerance (Siddiqui et al., 2015; Hu et al., 2021). There are some reports about the role of proline in nano-enabled salt tolerance. For example, nano-SiO_2_ can improve plant salt tolerance mainly by increasing proline content, photosynthetic rate, and water use efficiency of plant leaves (Siddiqui et al., 2015). Titanium dioxide NMs-treated plants not only induced the increase of antioxidant enzyme activity but also increased the content of proline and soluble sugar to improve osmotic balance in cells (Abdel-Latef et al., 2017). (3) Ascorbic acid plays an important role in plant growth and development (Zhu, 2002). After plants are subjected to abiotic stress, ascorbic acid can alleviate oxidative damage by maintaining ROS homeostasis and participating into the ASA-GSH cycle (Zhang et al., 2012). For example, exogenous nano-silicon can alleviate the damage of salt stress to soybean seedlings by increasing ascorbic acid content and enhancing antioxidant enzyme activity (Farhangi-Abriz and Torabian, 2018). Other studies showed that Cu NPs (Pérez-Labrada et al., 2019) and SiO_2_ nanoparticles (Pinedo-Guerrero et al., 2020) improved tomato salt tolerance *via* increasing ascorbic acid contents. Furthermore, it is well known that phenols and anthocyanins are also key members of non-enzymatic antioxidant system (Pinedo-Guerrero et al., 2020). ZnO NPs can increase the content of total phenols and anthocyanins in potatoes (Raigond et al., 2017). Adding copper nanoparticles (CuNPs) to chitosan–polyvinyl alcohol hydrogel (Cs-PVA) can also significantly increase the content of phenols, β-carotene, ascorbic acid, and lycopene in tomato, finally improving tomato salt tolerance (Hernández-Hernández et al., 2018a,b).

Besides modulating antioxidant enzymes and no enzymatic pathways, nanomaterials can also be used as delivery tool to deliver antioxidants to regulate plant salt tolerance. Chloroplast guiding peptide modified β-cyclodextrin conjugated quantum dots are able to do targeted delivery of methyl viologen and ascorbic acid to chloroplasts to regulate its redox status (Santana et al., 2020). Calcium-induced cross-linked pea protein nanoparticles can stably deliver the antioxidant resveratrol (Yf et al., 2020). The combination of resveratrol and α-tocopherol significantly improved the salt adaptability of citrus seedlings (Kostopoulou et al., 2014). Overall, use of nanomaterials to maintain ROS homeostasis through either direct ROS scavenging, or modulating antioxidant system, or delivery of antioxidants, could be an alternative way to improve plant salt tolerance.

## The Importance of Maintaining Na^+^/K^+^ Homeostasis for Plant Salt Tolerance

Maintaining Na^+^/K^+^ homeostasis is a hallmark for plant salt tolerance (Alsaeedi et al., 2018; Zhang et al., 2020; Liu et al., 2021a). Under salinity stress, plants evolved fine mechanisms to avoid over-accumulation of Na^+^ and massive loss of K^+^ to maintain Na^+^/K^+^ homeostasis. Shoot Na^+^ exclusion, root Na^+^ extrusion, and vacuolar Na^+^ sequestration are the main strategies for plants to avoid over-accumulation of Na^+^ in cytosol and thus the resulted Na^+^ toxicity (Yue et al., 2012; Flowers and Colmer, 2015; Wu, 2018a; Wu et al., 2018b, 2019, 2021; Zelm et al., 2020; Shah et al., 2021). For example, Wu et al. reported that vacuolar Na^+^ sequestration in the mature root zone might be responsible for the stronger salt tolerance of bread wheat than durum wheat (Wu et al., 2018b). Interestingly, previous studies showed that in root, the vacuolar Na^+^ sequestration was more important than Na^+^ exclusion for salinity tolerance in barley (Flowers and Colmer, 2015). These results suggest that the employed mechanisms for salt tolerance might be differed at tissue level in plant or between different plant species. No doubt, avoiding Na^+^ over-accumulation is also an important mechanism for nano-enabled plant salt tolerance. It has been reported that nanomaterials such as PNC (poly acrylic acid-coated cerium oxide nanoparticles) can enhance shoot Na^+^ exclusion to improve salt tolerance of cotton (Liu et al., 2021b). Also, cerium oxide nanoparticles shorten the apoplast barrier of *Brassica* roots to transport more Na^+^ to shoot, thus reducing the accumulation of Na^+^ in plant roots to improve its salt tolerance (Rossi et al., 2017). Other studies showed that silica nanoparticles can improve germination and growth of cucumber by decreasing Na^+^ content and maintaining K^+^/Na^+^ ratio under salinity stress (Alsaeedi et al., 2018).

Potassium plays important role in plant cell activities, i.e., adjusting of cell osmotic potential and charge balance, acting as a cofactor of many enzymes such as malate dehydrogenase and pyruvate kinase, promoting sugar transport and water retention of cells, and controlling of stomatal movement (Osakabe et al., 2013). Plants’ ability to maintain root and mesophyll K^+^ is known as important mechanisms for plant salt tolerance (Chen et al., 2005; Wu et al., 2013, 2014, 2015, 2018d). Not surprisingly, improving the ability to maintain K^+^ in plants is also a mechanism involved in nano-enabled plant salt tolerance. For example, through scavenging of ROS, cerium oxide nanoparticles modulate ROS-activated NSCC channels (non-selective cation channels) to reduce K^+^ loss to improve salt tolerance in *Arabidopsis* (Wu et al., 2018c). Similar results were also found in cotton (Liu et al., 2021b) and rapeseed (Khan et al., 2021). The foliar-applied poly(acrylic) acid-coated cerium oxide nanoparticles can promote shoot K^+^ retention and Na^+^ exclusion but not vacuolar Na^+^ sequestration to maintain Na^+^/K^+^ ratio to improve cotton salt tolerance (Liu et al., 2021b). It showed that nanoceria modulated the relative expression level of *HKT1* (upregulation) and *KOR* (downregulation) genes and showed no effects on the relative expression level of *NHX1* gene. This is in accordance with the findings of subcellular distribution of Na^+^ and K^+^ dye signals between control plants and nanoceria-treated cotton plants under salinity (Liu et al., 2021b). Nanosilica (SiNPs) treatment can increase K^+^ content of cucumber seedlings under high salt stress, thus improving cucumber salt tolerance mainly by maintaining K^+^/Na^+^ ratio (Alsaeedi et al., 2019). In addition, MWCNTs were also found to increase the transcriptional abundance of Na^+^ and K^+^ transporters through NO (nitric oxide) participation to maintain K^+^/Na^+^ ratio to increase rapeseed salt tolerance (Zhao et al., 2019). Taken together, maintaining Na^+^/K^+^ homeostasis might be a commonly employed mechanism for nano-enabled plant salt tolerance.

## The Role of Phytohormone in Plant Salt Tolerance

Plant hormones and plant growth regulators are essential for plant growth and development, especially in regulating plant response to stress (Hou et al., 2016; Zhu, 2016; Yu et al., 2020; Zhao et al., 2020). Abscisic acid (ABA), gibberellin (GA), brassinosteroids (BR), jasmonic acid (JA), and salicylic acid (SA) are common hormones that play a vital role in crop salt stress response (Zhu, 2002; Yu et al., 2020). However, its role in nano-enabled plant salt tolerance are not well explored. How phytohormones was involved in nano-enable plant salt tolerance and the possible effect of nanomaterials on plant growth-related processes under salinity stress are still largely unknown. Regarding the role of these hormones in plant resistance to abiotic stress such as salinity stress and plant growth-related processes, some good review papers are available (Huang et al., 2017; Wang et al., 2020; Yu et al., 2020).

### Abscisic Acid

Abscisic acid (ABA) is known as a stress responsive hormone. Its content was rapidly increased when plants faced to abiotic stress such as saline-alkali stress, water stress, and temperature stress (Liang et al., 2017; Yu et al., 2020). Besides modulating water absorption and proline accumulation, ABA improves plant salinity resistance by inducing the expression of salt tolerance genes (Zhao et al., 2020; Wu et al., 2021). The increase of ABA content under salt stress also maintains the stability of DELLA protein (a class of protein with the N-terminal having highly conserved DELLA domain), which attenuates cellular activity by regulating gibberellin and finally alleviates the damage of salt stress to plants (Lei et al., 2020). Generally, the increase of ABA content in plants under salt stress is positively correlated with its stress tolerance (Danquah et al., 2014; Zhu, 2016). Exogenous application of ABA always can alleviate plant salt stress symptom (Wei et al., 2015; Lei et al., 2020). Previous study showed that Ag nanoparticle can increase ABA content to improve plant abiotic stress tolerance (Khan and Bano, 2016b). Another study showed that nanopriming can improve germination percentage and germination rate of two rapeseed cultivars under salt stress by increasing the antioxidant enzyme activity and abscisic acid content (El-Badri et al., 2021). Furthermore, researchers used mesoporous silica nanoparticles to deliver ABA to improve drought tolerance in *Arabidopsis* (Sun et al., 2018).

### Gibberellins and Brassinosteroids

Gibberellins (GA) not only play an important role in promoting plant growth, inducing flowering and breaking dormancy, but also participate in plant response to abiotic stress (Daviere and Achard, 2013). Carbon nanotubes (e.g., SWCNTs) can promote the growth of seedlings by increasing the GA content in rice (Zhang et al., 2017). CeO_2_ NPs can enhance rice tolerance to N-deficiency by regulating antioxidant enzyme system and the levels of phytohormones including IAA, GA and ABA (Wang et al., 2020). Brassinoids (BR) is known as the sixth hormone in plants, having the role of promoting cell elongation and division and improving tolerance to salinity, drought and heat stresses (Zhu, 2002; Flowers and Colmer, 2015; Hou et al., 2019). BR can alleviate the inhibition of salt stress on rice seed germination and seedling growth (Anuradha and Rao, 2003). However, to date, the role of BR in nano-enabled plant salt tolerance is still obscure. How nanomaterials modulate BR biosynthesis and its signaling pathways to improve plant salt tolerance are worthy to be investigated in future studies.

### Jasmonic Acid and Salicylic Acid

Jasmonic acid (JA) and salicylic acid (SA) are main plant growth regulators in response to stress (Bernstein, 2019). Jasmonic acid is known to improve plant stress tolerance (Parvaiz et al., 2016). For example, under salinity stress, the level of JA content is positively correlated with salt tolerance in wheat (Zhao et al., 2014). Overexpression of JA related gene *TaAOC1* promoted the accumulation of JA in *Arabidopsis* leaf and improved plant salt tolerance (Zhao et al., 2014). TiO_2_ NPs have been shown to activate the JA pathway in wheat (Jiang et al., 2017). Silica nanoparticles can improve salt tolerance of rice by regulating jasmonic acid signal (Abdel-Haliem et al., 2017). In addition, some studies have shown that chitosan activates the octadecanoic acid pathway of JA and protects plants from salt stress by regulating cell ion concentration (Pichyangkura and Chadchawan, 2015). Adding Cu NPs to chitosan hydrogel can reduce the activation of JA gene expression under salt stress (Hernández-Hernández et al., 2018a,b).

Similar to JA, SA is a well-recognized hormone which can improve plant stress tolerance. Exogenous application of SA can significantly improve salt stress tolerance in crops such as rice (Jini and Joseph, 2017) and potato (Faried et al., 2017). SA induced salt stress tolerance is mainly associated with enhancing the activities of SOD and CAT and other antioxidant enzymes to alleviate over-accumulation of ROS, or promoting lateral root growth, or executing synergistic action with other hormones (Blumwald, 2000; Shaki et al., 2018; Abdoli et al., 2020; Yu et al., 2020). Addition of nano Fe_2_O_3_ during exogenous spraying of SA can significantly improve K^+^ content, Fe content, endogenous level of SA, and antioxidant enzyme activity in *Trachyspermum AMMI* L. and thus can improve its salt tolerance (Abdoli et al., 2020). More studies are encouraged to investigate the role of JA and SA in nano-enabled plant salt tolerance.

Together, it suggests that nano-enabled plant salt tolerance is associated with modulating phytohormones, although the employed mechanisms might be varied with nanomaterials or in plant species. Also, exploring nanomaterials as tool to do efficient and targeted delivery of phytohormones to modulate plant salt tolerance could be a direction for future studies. More efforts are encouraged to study the role of phytohormones in nano-enabled plant salt tolerance and to better use phytohormones as plant growth regulators *via* nano-biotechnology.

## Molecular Mechanisms Underlying Plant Salt Tolerance

Abscisic acid (ABA) signal transduction pathway, protein kinase pathway, and salt overly sensitive (SOS) signal transduction pathway are the common and well-studied signal pathways of plants in response to salt stress (Zhu, 2002; Shaki et al., 2018). Here, the discussion will be focused more on these pathways.

### ABA Signal Transduction Pathway

The ABA pathway can be classified as ABA-dependent and ABA-independent pathways. The expression level of ABA-dependent genes is associated with the content of ABA *in vivo*. The expression of ABA-independent genes is affected by external environmental factors such as saline-alkali, drought, and temperature (Xiong et al., 2001). ABA receptor proteins PYR/PYL/RCARs (pyracbactin resistance/pyracbactin resistance-like/regulatory component of ABA receptor), PP2C (protein phosphatases type 2Cs), and SnPK2s (Sucrose non-fermenting 1-related protein kinases subfamily 2) act as core proteins for ABA signaling pathway (Ben-Ari, 2012). Under salt stress, ABA receptor protein PYR/PYL/RCARs sense ABA signals and bind ABA to inhibit protein phosphatase PP2C, which in turn enhance protein kinase SnRK2s activity, thus conveying ABA signals to downstream targets (Raghavendra et al., 2010). Reversible phosphorylation of proteins is also an important step in ABA signaling pathway. Protein kinases such as CDPKs (Calcium-Dependent Protein Kinases) and SnPKs (Sucrose non-fermenting-1-related protein kinases) positively regulate the expression of downstream genes in the ABA signaling pathway. However, they are negatively regulated by protein phosphorylases ABI1 and ABI2 (Lim et al., 2012). The ABA signaling pathway also coordinated with various calcium signaling systems to respond to salt stress in plants. To date, less attention was paid to unveil the involvement of ABA signaling pathway in nano-enabled plant salt tolerance.

### Protein Kinase Pathways

The protein kinase pathways mainly include mitogen-activated protein kinase (MAPK) cascade pathway and calcium-dependent protein kinases (CDPK) cascade pathway (Chen et al., 2021). Both of the protein kinases are serine/threonine protein kinases which are widely distributed in plants (Chen et al., 2021). The MAPK cascade signals are mainly transmitted to downstream proteins through phosphorylation and dephosphorization and finally activate the expression of relevant stress-resistant genes to respond to plant abiotic stress (Nakagami et al., 2005). MAPK pathway executes signal transduction through sequential phosphorylation of three serine/threonine phosphoprotein kinases: MAPKKKs, MAPKKs, and MAPKs (Danquah et al., 2014). Overexpression of *OsMAPK5* and *OsMAPK44* genes in rice significantly alleviate the negative effect of saline stress to plants (Jeong et al., 2006; Xie et al., 2012). Overexpression of *ZmMKK1* and *ZmMKK4* genes in maize can improve salinity tolerance of transgenic *Arabidopsis thaliana*, and overexpression of *GhMPK2* and *GhMAP3K40* genes improve cotton salt tolerance (Kong et al., 2011; Liang et al., 2011; Chen et al., 2015). Similarly, less attention was paid to the possible modulation of protein kinase activities in nano-enabled plant salt tolerance.

As a second messenger, calcium plays a central role in plant cell signal transduction. Ca^2+^ signal sensing proteins in plants mainly include calmodulin (CaM), calmodulin-like protein (CMLs), calmodulin B-like protein (CBL), and calcium-dependent protein kinase (CDPKs; Zhu, 2016; Cao et al., 2020). For example, CaM is the most widely distributed and important calcium-dependent protein kinase, and CDPKs are unique serine/threonine protein kinases in plants (Kim et al., 2013). These Ca^2+^ signal sensing proteins work together to form a large signal transduction regulatory network and transmit Ca^2+^ signals to downstream response elements (Batisti and Kudla, 2012). Ca^2+^ signaling pathways also play a vital role in nanoceria-induced response to salt stress (Brahimova et al., 2021).

### SOS Signal Transduction Pathway

SOS signal transduction pathway is an important way for plants to maintain Na^+^ homeostasis under salt stress and mainly executed by three kinds of proteins: SOS1, SOS2, and SOS3 (Yue et al., 2012; Zhu, 2016). The general process of SOS signal transduction is: under salt stress, intracellular Ca^2+^ concentration increases rapidly, the SOS3 (the upstream Ca^2+^ binding protein) and SCaBP8/CBL10 (SOS3-LKE calcium-binding protein8/calcineurin B-like Protein10) sense Ca^2+^ signals and interact with SOS2 to form SOS3-SOS2 protein kinase complex to regulate SOS1 activity to exclude Na^+^. Studies have shown that SOS2 interacts with CAT2 and CAT3, and the SOS signal transduction pathway may also coordinate with ROS signal to participate in plant salt stress response (Verslues et al., 2007). Singh et al. reported that iron oxide nanoparticles can enhance salt tolerance of trees by increasing the expression levels of genes such as *HKT1*, *SOS1*, and *NHX* and the activity of antioxidant enzymes (Singh et al., 2021). Similarly, Liu et al. found that nanoceria treatment upregulated the relative expression level of *HKT1* (shoot Na^+^ exclusion) but not SOS1 to improve cotton salt tolerance (Liu et al., 2021b). The mechanisms of nanomaterials on maintaining Na^+^ homeostasis in salt-stressed plants are different in nanomaterials and also in plant species.

### Transcription Factors and Stress-Responsive Related Genes

Transcription factors such as WRKY (a class of protein with the N-terminal having highly conserved WRKYGQK domain), NAC [NAM (no apical meristem, *Petunia*), ATAF1-2 (*Arabidopsis thaliana* activating factor), and CUC2 (cup-shaped cotyledon, *Arabidopsis*)], bZIP (basic leucine zipper), and AP2/ERF (APETALA2/Ethylene Responsive Factor) play an important role in plant response to salt stress. WRKY is a plant-specific transcription factor and also responds to biotic and abiotic stresses. Overexpression of *WRKY25* or *WRKY33* genes can improve salt tolerance of *Arabidopsis thaliana*, and the double mutant plants of *wrky25* and *wrky33* are more sensitive to salt stress (Ding et al., 2014). Plant-specific NAC transcription factors are also play a role in abiotic stress response (Ding et al., 2014). Overexpression of *OsNAC2*, *OsNAC6*, and *OsNAC045* genes in rice significantly improved salt and drought tolerance (Ohnishi et al., 2005; Hu et al., 2010; Xiang et al., 2020). However, *AtNAC2* overexpressed plants are more sensitive to salt stress, indicating the complexity of NAC transcription factors in response to plant abiotic stress (Balazadeh et al., 2010). Basic leucine zipper (bZIP) protein was widely involved in plant stress response. bZIP transcription factors participate in ABA signal transduction pathway and regulate the expression of related abiotic stress responsive genes. Overexpression of *AtbZIP1* improved salt tolerance in *Arabidopsis* (Sun et al., 2012). Furthermore, *AtERF98* gene can improve the tolerance of *Arabidopsis* to salt stress by promoting the synthesis of ascorbic acid (Zhang et al., 2012). Plants with *GmERF3* gene overexpression showed better salt and drought tolerance, and significantly higher contents of proline and soluble sugar than control plants (Zhang et al., 2009).

Moreover, the expression of genes involved in osmotic regulation, ion balance, antioxidant, and hormone regulation will also change after plants are subjected to abiotic stress such as saline-alkali stress (Cao et al., 2020; Zhang et al., 2020). For example, the expression level of *HKT1* (high-affinity K^+^ transporter for Na^+^ exclusion) gene was significantly increased in PNC treated cotton seedlings compared with the non-PNC treated seedlings under salt stress (Liu et al., 2021b). NHX1 (Na+/H+ exchanger 1), a Na^+^/H^+^ antiporter gene, plays an important role in Na^+^ compartmentalization. Overexpression of *AtNHX1* gene showed improved salt tolerance in wheat (Xue et al., 2004), cotton (He et al., 2005), and soybean (Li et al., 2010). DELLA protein is a vital negative regulator of the gibberellin signaling pathway and also plays an important role in other hormone signaling and environmental signaling systems (Zhu, 2002). The higher content of DELLA protein in *Arabidopsi*s, the stronger its salt tolerance (Zhu, 2002). Halophytes can promote the accumulation of DELLA protein by inhibiting GA signal transduction to prolong the static growth period of plants to improve their salt tolerance (Murase et al., 2008). DELLA protein can also improve the activity of SOD and CAT in *Arabidopsis* and wheat under salt stress and enhance the ability of scavenging ROS in plants, suggesting that the overexpression of the *DELLA* gene can significantly improve plant salt tolerance (Murase et al., 2008; Dobrikova et al., 2017).

## Conclusion and Perspectives

Nowadays, nanomaterials showed potential in improving plant salt tolerance. However, the relevant mechanisms need to be further explored. Also, to date, nano-enabled plant salt tolerance is still largely demonstrated at the laboratory research stage. To facilitate the adoption of nano-enabled plant salt tolerance in agricultural production, discussions and setup of widely accepted policies and regulations are urgently called on task. Also, more studies should be conducted to explore the possible effect of nanomaterials on plants under salt stress from the viewpoint of source-sink regulation. For example, if nanomaterials are foliar-sprayed to plants, its effects on sink capacity should be studied. Studies have shown that Zn chitosan nanomaterials significantly increased the accumulation of starch biosynthetic enzymes in wheat grains and thus the yield (by 21%) compared with the control group treated with ZnSO_4_, which further verified that the wheat treated with nanomaterials on the leaf had better sink strength (Kumar et al., 2021). Overall, we believe that nanotechnology can play an important role in sustainable development of agriculture.

## Author Contributions

HW and ZZ conceived this review paper. ZL and LZ summarized and analyzed literatures regarding mechanisms underlying plant salinity stress tolerance and nano-enabled plant salt tolerance. FZ and JL contributed to make the tables and figures. XZ and XK contributed to the discussion of manuscript. HW, ZZ, ZL, and LZ wrote the manuscript. All authors contributed to the article and approved the submitted version.

## Funding

This work was supported by National Natural Science Foundation of China (32071971, 31901464), joint project SZYJY2021008 from Huazhong Agricultural University and Agricultural Genomics Institute at Shenzhen, Chinese Academy of Agricultural Sciences, and project 2662020ZKPY001 supported by the Fundamental Research Funds for the Central Universities to HW and the Key Science and Technology Special Project of Xinxiang City of China (ZD2020004), Leading Talent Project in Science and Technology Innovation of Central Plain of China (214200510021), and the Program for Innovative Research Team (in Science and Technology) in the University of Henan Province (21IRTSTHN023) to ZZ.

## Conflict of Interest

The authors declare that the research was conducted in the absence of any commercial or financial relationships that could be construed as a potential conflict of interest.

## Publisher’s Note

All claims expressed in this article are solely those of the authors and do not necessarily represent those of their affiliated organizations, or those of the publisher, the editors and the reviewers. Any product that may be evaluated in this article, or claim that may be made by its manufacturer, is not guaranteed or endorsed by the publisher.
